# Piloting the UK’s First Home-Office-Licensed Pharmacist-Led Drug Checking Service at a Community Substance Misuse Service

**DOI:** 10.3390/bs10080121

**Published:** 2020-07-25

**Authors:** Amira Guirguis, Rosalind Gittins, Fabrizio Schifano

**Affiliations:** 1 Swansea University Medical School, Institute of Life Sciences, Swansea University, Swansea SA2-8PP, Wales, UK; 2 Psychopharmacology, Drug Misuse and Novel Psychoactive Substances Research Unit, University of Hertfordshire, Hatfield AL10-9AB, UK; f.schifano@herts.ac.uk; 3 Humankind Charity, Inspiration House, Unit 22, Bowburn North Industrial Estate, Durham DH6 5PF, UK

**Keywords:** drug checking, harm reduction, Home Office, Raman spectroscopy, substance misuse, overdose, opiates, opioids, cannabinoids, drug testing, pill testing

## Abstract

(1) Introduction: Drug-related deaths in the UK are at concerning high levels. The unknown content and purity of illicit substances can cause unpredictable adverse effects and thus a public health risk with no sign of abating. On-site drug checking is a public health strategy that has previously been implemented, predominantly in festival settings, but without Home Office licensing. (2) Aims: The aim of this study was to pilot the UK’s first pharmacist-led, Home Office-licensed community drug checking service. (3) Methods: A bespoke protocol incorporating legally, professionally and ethically binding documents was implemented. This free, confidential service ran between February and March 2019, was available to anyone over 18 who were purposefully recruited, gave informed consent and agreed to relinquish their drug sample. Samples were checked on-site within an established Substance Misuse Service (SMS) using a handheld Raman spectrometer to determine likely drug content and adulterants. In parallel, participants completed a questionnaire about their substance use and the drug sample(s) being tested. A pharmacist-led multidisciplinary approach was adopted to discuss the analytical findings. Informed by the results of the analysis and the questionnaire, people who used the service received tailored harm reduction advice. (4) Results and Discussion: The pilot operated for a total of four days over four weeks. Eleven people visited and relinquished a total of thirteen samples. Half of the participants had previously overdosed and were known to the SMS. Seventy per cent were male, all were White British individuals, 30% were employed and two people disclosed visiting from another nearby town. Samples included what was thought to be heroin, synthetic cannabinoids, stimulants, benzodiazepines and LSD and none required activation of the “alerts cascade” process. Most participants drank alcohol regularly and the concomitant use of traditional illicit drugs and prescribed medication (including opioids, anxiolytics and antidepressants) with sedating profiles was common. Given some of the ethical decisions and interpretation of the results, specialist pharmacist involvement was deemed essential. (5) Conclusions: This pilot demonstrated the proof-of-concept that a pharmacist-led Home Office-licensed drug checking service can be successfully implemented in community SMSs.

## 1. Background 

Over the last two decades, the drug market has dramatically changed, posing unpredictable public health risks and a significant burden on national health and social care services. In England and Wales, the number of deaths related to substance misuse has increased by 3.5-fold from 831 in 1993 to 2917 deaths in 2018, a 16% increase from the previous year [1]. The illicit market has become increasingly complex, particularly with the global emergence of New/Novel Psychoactive Substances (NPS), drug use trends, easy online access to illicit substances, peer reviews and limited technologies for the identification and monitoring of novel substances. In response to these challenges, harm reduction interventions are essential to promote public health and prevent avoidable premature deaths from substances [2,3]. The International Harm Reduction Association defined “harm reduction” as “policies, programmes and practices that aim primarily to reduce the adverse health, social and economic consequences of the use of legal and illegal psychoactive drugs without necessarily reducing drug consumption” [2].

Lefkovits (2016) proposed various harm reduction interventions. These include on-site drug checking, monitoring through establishing an early warning system and publishing police seizure data. The latter is not widely implemented as it may be viewed as promoting drug use [4]. In contrast, the former “drug checking”, also known as “pill testing”, “street drug analysis”, “adulterant screening”, “multi-agency safety testing” and “drug safety testing” [5,6,7], is a model of harm reduction that can be provided at the point of care. Drug checking involves drug analysis to identify the content and/or purity of the drug sample, then the provision of analysis results are directly communicated to individuals through a harm reduction consultation, an online report and/or official channels to cascade alerts from a potentially lethal substance [7,8]. A global review was undertaken in 2017 and identified 31 drug checking services in 20 different countries [6,9]. In 2018, Oute et al. also carried out a comprehensive review of drug checking services based within the nighttime economy [8]. An updated review of drug checking services was carried out by Guirguis et al. (2020) [10]. In these reviews, established drug checking services were compared with respect to their impact and limitations. In addition, the latter review captured the general public’s perceptions on drug checking via Twitter [10].

Although there are claims that drug checking lacks a robust evidence base regarding its effectiveness [3,8,11], based on Public Health England’s “drug alerts—evidence for effectiveness”, drug checking has been proven to be effective in reducing use [12], including “harmful use and limiting specific substance use amongst certain user groups”. Streetwork, the youth advisory service in the city of Zurich, evaluated their on-site and stationary drug checking facilities, demonstrating that drug checking coupled with a consultation contributes to harm reduction and prevention, particularly for those individuals with frequent re-dosing patterns and polysubstance use [13]. Although not all drug checking techniques are conducted to a “forensic standard”, they have been shown to reduce risky consumption behaviours [11,14]. Drug checking has also been shown to enable engagement of individuals who are not in treatment [15] and has provided drug education focusing on “safer drug use” [16]. It is also claimed that drug checking services can “shift and stabilise” the drug market since individuals can consequently make more informed decisions related to the drugs they are buying and/or intend to consume [11]. Drug checking services have obvious positive effects. These positive effects include: (1) reducing harms from drugs by enabling a dialogue through consultation and counselling about their consumptions patterns and drug-taking behaviours; (2) enhancing the monitoring of drug trends through drug analysis; (3) monitoring of the emergence of novel substances; (4) improving access to healthcare and substance use treatment services; and (5) improving sharing of information with relevant stakeholders [5,8,13,17]. However, drug checking has been a “controversial” harm reduction activity and may have negative effects by implying the normalisation and safety of drug use and limited accuracy of findings, for example by overestimating drug concentrations [3,8,18].

The first drug checking service was established in the early 1990s in Europe to reduce harms from NPS, and the first drug checking service that combined drug testing with harm reduction interventions was Drug Information and Monitoring System (DIMS) established in the Netherlands in 1992 [19]. Typically, “drug checking services” invite recreational users to anonymously submit a sample of an illicit substance for analysis and aim to deliver harm reduction advice to the person who provided the sample, based on the sample’s identified content (type of drug(s) and/or purity) [6,8]. Drug checking can be “front-of-house” or “back-of-house” testing [7]. The former refers to a service where individuals attend the on-site drug checking service, at festivals for example, and personally engage with the team conducting the service. It usually targets people who use substances and aims at reducing harms in the nighttime economy [7,8]. In contrast, the latter refers to a service, where individuals may anonymously send drug samples for analysis and the results may be published on the service’s website to raise awareness about a drug type [7]. Back-of-house does not usually involve a direct interaction between the team conducting the service and the individual bringing a drug sample for analysis. It may also involve drug samples from police seizures. Back-of-house predominantly aims at monitoring trends in the drug market and providing wider non-targeted harm reduction messages [7,8]. Drug checking may be on-site providing a “screening” grade analysis or laboratory-based providing “confirmatory” analysis. This primarily depends on the technologies employed and available resources, which in turn can significantly impact associated costs, the analysis time and the quality of the results [4,11,20]. 

Drug checking services have taken place at a variety of settings including within the nighttime economy, music festivals, shopping centres and in laboratories. In the UK, in 2009, WEDINOS (Welsh Emerging Drugs & Novel Substances Project) was the first service to collect unknown substances from substance misuse services (SMS), housing and hostels, youth clubs and young people’s services, night clubs and bars, mental health services, Local Authorities, Ambulance Service and the Police. WEDINOS tests the substances in laboratories using confirmatory analysis techniques, produce and disseminate pragmatic harm reduction advice, based on the content and legal context, via their website, health alerts via press releases and their quarterly bulletin. Recently, The Loop has been testing drugs predominantly at UK music festivals using on-site screening techniques and has demonstrated the need for such services to be providing harm reduction advice in such settings [21]. WEDINOS operates under a Home Office licence and provides a free service, which is funded by the Welsh Government, whereas the latter is a volunteer-led service, which operates without a Home Office licence and, instead, in collaboration with relevant local police forces under “an exception to legal restrictions”. 

The technological and analytical capacity of the testing along with tailored counselling plays a key role in the success of a drug checking service. Numerous reports have compared and evaluated various screening and confirmatory technologies that are employed in drug checking services [4,6,8,11,20]. These techniques include colorimetric reagent testing, Fourier-Transform Infrared Spectroscopy (FTIR), Capillary Electrophoresis (CE) with Ultraviolet Spectroscopy (UV), High-performance Liquid Chromatography (HPLC) with UV or Mass Spectrometry (MS) detection, Gas Chromatography with MS detection (GCMS), Ion Mobility Spectrometry (IMS) with MS detection and Matrix-assisted laser desorption ionisation (MALDI) Orbitrap MS [8,22]. These techniques vary in their ability to detect different substances, multiple compounds at once, low drug content in complex mixtures such as designer fentanyls, quantitative analysis, previously unknown substances, analysis time, portability, reliability, ease of operability, cost and suitability for a particular setting [4,6,8,11,20]. Despite that laboratory-based MS methods are the gold standard, presumptive techniques such as colour tests may enable initial harm reduction conversations. Given the limitations of in-field screening techniques with respect to false positive and false negative results, they can still help to engage people who use drugs in a dialogue about their consumption patterns, which can then lead to positive behaviour change. It is important to note that the cost of operation is very high, and that gold standard methods may not be viable for these services. 

In addition, the choice of the technology depends on the purpose of the analysis, for example, whether the analysis aims at identifying the psychoactive substance only or whether the analysis aims at identifying all the constituents in a drug sample or providing quantitative analysis. The quality of the outcomes not only depends on the suitability of the technology but also on the personnel undertaking the analysis and their level of expertise. The quality of the drug checking is enhanced not only by the drug checking activity itself but also by the use of the results, i.e., whether the results will be used to provide individual harm reduction intervention(s), issuing public health alerts, disseminating harm reduction information and/or surveillance of the drug market [11]. Harper et al. (2017) recommended Infrared and Raman as the best technology for on-site drug checking. In this paper, evaluation of the technology employed for the analysis of drug samples is not discussed and will be thoroughly covered in a subsequent analytical paper. 

Drug checking services are deemed important as drug-related deaths are at concerning levels [1]. In addition, the emergence of batches laced with potent and potentially harmful substances such as Spice and fentanyl derivatives continue to affect marginalised and vulnerable groups, with no sign of abating [23,24]. The frequency, variety and chemical diversity of newly available substances as well as their unpredictable adverse effects are posing acute public health risks [25]. These risks are particularly evident for NPS, as over 900 new substances have emerged in the last decade, thus making drug use more dangerous and communicating accurate harm reduction advice even more challenging [25].

This paper presents learnings from a pilot service that follows the UK Government’s agenda to prepare and respond to future threats caused by NPS [26]. It is also in line with the Royal Pharmaceutical Society’s guidance documents developed by the authors of this manuscript [27]. We believe drug checking services are important harm reduction strategies. In the UK, drug checking services have never been undertaken in a clinical setting or coupled with clinical interventions or under a Home Office licence for field drug detection. Therefore, the aim of this work was to scope the feasibility of setting up and providing a pharmacist-led Home Office-licensed drug checking service in a community SMS environment using a portable screening laboratory. The project’s objectives were to engage individuals in treatment and provide tailored harm reduction interventions including provision of take home naloxone, injecting paraphernalia, psychosocial and prescribed treatment interventions, signposting individuals to appropriate sources of help and support and avoiding drug related harms by enabling more prompt responses to potential “bad batches” using existing alert cascade processes (including interface with Public Health England and NHS England Controlled Drug Local Intelligence Network protocols).

## 2. Research Question

Would this project, the first pharmacist-led, UK Home Office-licensed drug checking service embedded in an established SMS, be used by people who use drugs (to then eventually access more support services) and would it increase access to tailored harm reduction advice?

## 3. Methods

### 3.1. Site Selection

The pilot ran for four days over a four-week period at a community SMS (Addaction’s Weston-Super-Mare site in North Somerset), which is commissioned to provide SMS by the Local Authority and is regulated by the Care Quality Commission (CQC). This site was selected because it was an established and integrated SMS, with approximately 700 registered active clients and an additional 75 who were accessing the needle syringe provision scheme. 

### 3.2. Patient and Public Involvement (PPI)

To inform the study design, a PPI study was conducted. The study involved discussions or free chats with clients who randomly attended some of the regular group activities at the clinic. For this study, verbal consent was obtained to anonymously capture clients’ views on the “idea” of the proposed service as well as particulars of the methodology, e.g., using rewards such as vouchers to strengthen the recruitment process. Therefore, people who were known to the SMS (*n* = 30) were consulted, by the principal investigator, prior to the start of the study to capture their views on whether they would engage with such a service and evaluate their acceptability of whether they would be willing to relinquish a drug sample for the purpose of drug checking. These included three groups (the women group and two mixed groups at different stages of recovery) who attended the service for regular group support. Staff (*n* = 6) including recovery workers, and people in recovery were also consulted. The PPI study showed acceptability of the proposed service but also highlighted reasons for possible disengagement including fear of being made known to the police if reward vouchers were used. Therefore, during this pilot drug checking service, no reward was provided to encourage participation.

### 3.3. Stakeholders

Several stakeholders proactively engaged with the pilot, including local commissioners, police, people accessing the SMS and staff. These stakeholders were consulted on their views to establish the feasibility and acceptability of the service.

### 3.4. Compliance with Legislative Requirements

To comply with current legislation [28], Home Office requirements, the General Pharmaceutical Council standards for pharmacy professionals and the SMS local and site policies (e.g., safeguarding and drug use on premises), site compliance developments were undertaken (e.g., relevant security requirements, health and safety, compliance with safe custody regulations, controlled drug records and relevant signage). In addition, a bespoke protocol was developed, which covered every aspect of the running of the service. Additional documents included the development of various legally, professionally and ethically binding documents, including a site Home Office licence, a statement of intent, ethics approval by both organisations (the collaborators: Addaction and the University of Hertfordshire (UH)), participant information sheets, consent forms, rigorous health and safety assessments (treating any unknown sample as a potentially lethal fentanyl derivative) and a Memorandum of Understanding with various stakeholders and contributors. All partners and contributors involved in this pilot agreed that they do not condone the use of illegal drugs and that the safest way to take drugs is not to take drugs at all. Samples were not returned to their owners. They were handled in line with all legal requirements under the Home Office licence: they were manipulated for checking, coded, sealed in a labelled evidence bag, entered into a Controlled Drug Register and stored in a controlled drug cabinet, which met The Misuse of Drugs (safe custody) regulations 1973. 

### 3.5. Staff Training and Operational Delivery Including Sample Recruitment

**Figure 1:**
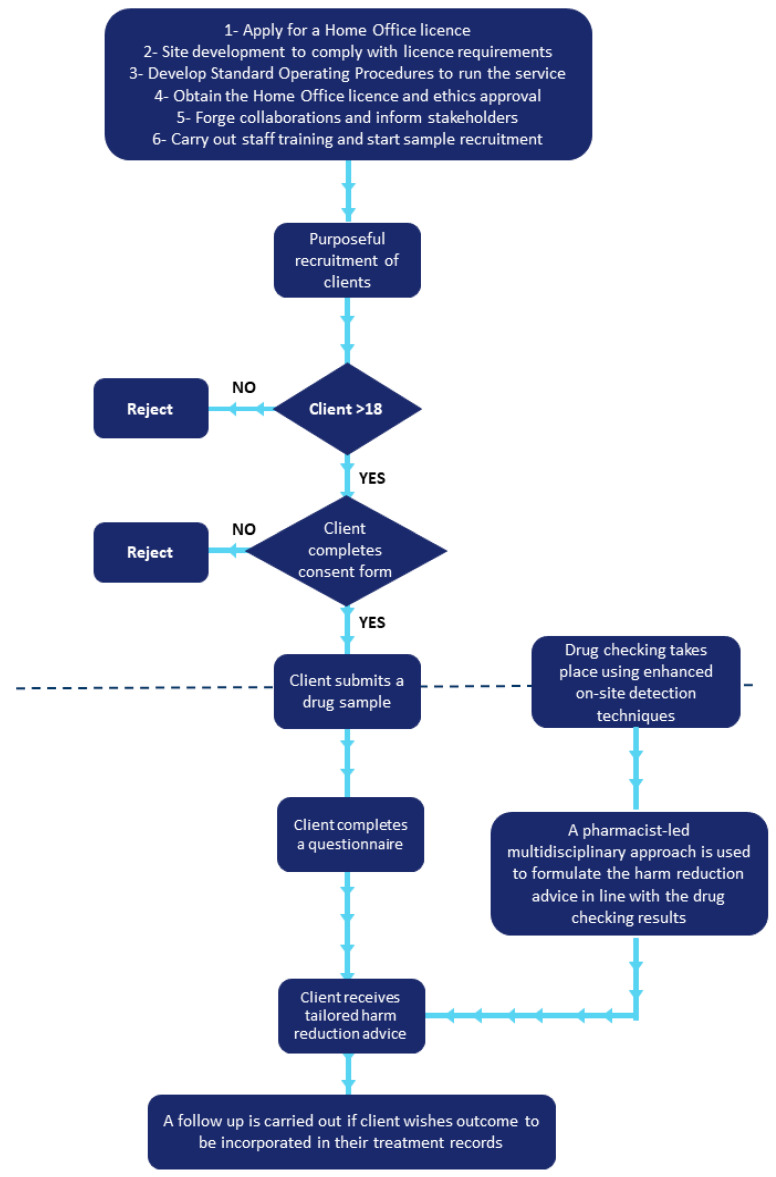
A summary of the study process.

The SMS (Addaction) staff based at the selected site were trained by the project leads on the step-by-step running of the new service using the bespoke protocol. The protocols were shared with involved staff members prior to the pilot day to ensure they were well prepared for the training day and to address any questions or concerns. The face-to-face training day (18 February 2019) involved an oral presentation on the service and its aims, methodology and importance in harm reduction. It also included a trial run of the service to ensure staff were aware of maintaining confidentiality of the individuals who will use the service and compliance with legislative requirements. The Loop contributed to additional virtual training (via Skype) to the SMS Staff on how to communicate the results to the people who accessed the service. In addition to discussions with the Home Office Compliance Team, the staff training day enabled the refinement of the protocols to ensure a smooth running of the service (see Figure 1). The service was piloted for a total of four days over a four week period (22 and 27 February 2019 and 6 and 15 March 2019). These days were selected to minimise any disruption to the day-to-day SMS operations.

The free, confidential service was available to anyone over the age of 18 who read the participant information sheet, gave informed consent and agreed to relinquish a sample of their substance. Participants could choose to be anonymous to the SMS; however, those who were prescribed clients were purposefully recruited by the clinic’s staff members. Recruitment started on the training day and continued until Day 4 of the study. People not known to the service attended the clinic after it first appeared in various media platforms (after Day 2 of the study). Recruitment was hampered by numerous barriers including local political elections. If the person was known to the SMS, their care was not affected, and the results of the checks remained confidential unless the person requested otherwise. The results of the checks were not recorded on the SMS’s clinical management system: identifiable information was coded so that the results were anonymised, processed and stored in line with General Data Protection Regulation (GDPR) and ethical requirements (University of Hertfordshire ethics protocol reference: aLMS/SF/UH/03459).

The service was designed as a collaboration between the University of Hertfordshire and a community SMS (Addaction’s Weston-Super-Mare site in North Somerset). The University of Hertfordshire (Principal Investigator) undertook on-site drug checks using a portable point-of-care screening technique (handheld Raman spectroscopy) to determine the likely content of the drug samples in real time. In parallel, the owner of the substance was asked to complete a short questionnaire (see Appendix A) to allow preliminary evaluation of the service and the design of tailored harm reduction advice. The questionnaire consisted of 15 questions, was piloted among the clinic’s staff and was informed by the PPI discussions. This questionnaire covered “other” substances that participants were using (including prescribed medication), information about their physical and mental health, their demographic characteristics and their engagement with treatment of substance use disorders. Following a multidisciplinary team discussion, which included the results of the analysis and a review of the completed questionnaire, the best approach to tailor the harm reduction advice was formulated and delivered. To evaluate the impact of the harm reduction-focussed service, following the delivery of the intervention, individuals were asked if they would do anything differently as a result of the intervention. In addition, a follow up was offered to individuals who wished outcomes to be included in their treatment records.

### 3.6. Ethics

Ethics approval was by the Health, Science, Engineering and Technology Ethics Committee with Delegated Authority, University of Hertfordshire, Hatfield, UK (ethics protocol reference: aLMS/SF/UH/03459) and from Addaction’s internal governance process.

### 3.7. Samples

Individuals submitted a few milligrams to 300 mg of sample, a tablet or part of a tablet. Sometimes the submitted sample was in plastic bags or carefully wrapped in paper or cling film. 

### 3.8. Drug Analysis Method

Samples were directly analysed through their plastic packaging or a glass vial using handheld Raman spectroscopy. Powders in wraps were analysed through a cover glass. For complex mixtures (such as herbs or formulated tablets with potentially high cutting agent and low psychoactive substance content), drug samples were dissolved in an appropriate solvent to separate the cutting agent(s). The technology employed in this pilot was based on extensive research developed by the research group (University of Hertfordshire) to enhance detection of psychoactive substances from street drug mixtures [29,30,31,32]. Details of how this research was employed to enhance drug detection in this pilot service will be thoroughly discussed in a subsequent paper.

### 3.9. Drug Alerts Cascade

A drug alert cascade is a method of “cascading information” related to a novel substance or a substance that has been linked to severe harm to relevant public health and other organisations for the purpose of reducing health risks from these substances. In this project, the identification of potent substances such as designer fentanyls would warrant a drug alert cascade.

## 4. Results

The pilot project was conducted from the 22 February 2019 to15 March 2019 over a total of four days. Additionally, a trial “run through” with staff training was completed on 18 February 2019. Referring to Table 1, eleven people visited the service for drug checking and supplied a total of thirteen drug samples. Seventy per cent were males. All were White British individuals, 30% were employed and two people disclosed visiting from another nearby town. The age range of the participants was 28 to 55 years old except for one participant who did not disclose their true age. The samples were primarily for their own use, obtained from street dealers, family/friends and in one case online. No samples warranted activation of the “alerts cascade”.

For ethical reasons (where a drug sample was brought by a third party with a lack of consent from the drug user and underaged individual), drug checking and consultation were declined by the multidisciplinary team on a couple of occasions, and, in one case (#013), a parent who had “found” the sample did not have the results disclosed to them; instead, they consented to the information being shared with the local police. Safeguarding concerns were also raised and it allowed the opportunity for the parent to be provided with additional support and counselling. On another occasion, a 17-year-old (#017-8) attended, accompanied by an adult representative; however, it was only after the harm reduction intervention had started to be delivered that the truth about their use and their age was disclosed. At this point, informed consent was assessed, and the decision made to continue with the intervention due to the nature of the issues being raised and signposting to the appropriate young persons (including mental health) services was provided. There were occasions where the person requested that their recovery worker was present so that they could be involved in the discussion about the results. 

The majority of participants drank alcohol regularly and used other illicit substances concomitantly including heroin, cocaine, amphetamine, methamphetamine, ecstasy, cannabis and benzodiazepines, particularly diazepam and alprazolam (Xanax^®^) (Table 1). Concomitant use of prescribed medication was also common, including opioid substitutes, anxiolytics and antidepressants. Medication with sedating profiles were often prescribed and used with alcohol and other substances which may also cause drowsiness and respiratory depression [33,34]. Evaluation of the technology employed in drug checking and detailed analysis of supplied drug samples will be discussed in a subsequent paper (in preparation).

The harm reduction advice was tailored to the needs of individuals (Table 2) and included support with substance use, dependence, addiction, psychosocial and mental health issues. Interventions involved appropriate referrals to general practitioners (GPs) or other health and social care services. Harm reduction advice included discussions regarding the inherent risks associated with illicit substances, harms of bingeing on drugs and alcohol, engagement with treatment and detoxification, discussion of adverse effects that may result from drug–drug interactions such synergistic or additive effects, including QT prolongation. In addition, over half of the participants had previously overdosed and were known to the service; therefore, overdose awareness dominated the harm reduction advice provided.

With respect to people’s perceptions of the service, there were no incidents of violence or aggression whilst the service was in operation. The service was well received locally, nationally and internationally. People’s perceptions were positive: they reported that the service was an excellent idea. Even during this small pilot, people were supported to take safer and more informed decisions. They were given practical advice tailored to their needs, for example, how to cut down drug usage in a way that reduced the risk of side effects and withdrawal symptoms such as seizures. The team also raised awareness of blood-borne viruses and gave information about the vaccinations and testing that could be offered to them. A number of participants reported that, after discovering the likely content of their drugs, that they planned to take less and buy drugs less often. Some participants did not feel they needed or wanted more advice or access to treatment at that time but knew where to seek help in the future. The main aim was to ensure that participants experienced a warm welcome and a positive and non-judgmental service. It was evident that participants need to be enticed into treatment and that clinical outcomes needed to be flexible.

## 5. Discussion

In this paper, we report the findings and learnings from establishing the first pharmacist-led, UK Home Office licensed drug checking service embedded in an established SMS. The focus of this paper is to highlight the requirements of setting up a drug checking service that operates within a licence framework from the UK Home Office. The focus is also to highlight the governance strategies required to conduct the service under the supervision and leadership of pharmacists in a SMS setting. For the purpose of this pilot, the term “drug checking” was used as this is an accurate reflection of the service offered, and it highlights that the organisations involved in this pilot do not condone drug use, provide confirmatory analysis or state that substances are “safe” to use. “Drug testing”, “pill testing” and “pill safety testing” were terms often used to imply that purity information was reported to the drug user and that the sample may be safe to use.

As illicit substances continue to be linked to serious harm, including drug related deaths and as drug culture continues to evolve [13], drug checking services are deemed to be an important harm reduction tool and “a measure of selective prevention” [13]. These services require the transfer of knowledge about specific drugs between service providers and people who use substances [35] and establishes a communication channel to reduce risky behaviours related to drug usage. In recent years, NPS use has become prevalent among diverse cohorts including the homeless, prisoners, clubbers, high-risk drug users such as heroin-injecting users, MSM (i.e., men who have sex with men) and abstinence treatment entrants [36]. Our previous study has also demonstrated the use of NPS among individuals in treatment [37]. Therefore, the purpose of this pilot was to support individuals to make informed decisions about their drug use, reduce drug related harms and assist with the monitoring of drug trends and communication of drug alerts as found by other researchers in this field [6,38].

The patient and public involvement (PPI) study undertaken amongst key stakeholders, including people who were accessing the SMS, prior to the start of the pilot, showed that they were receptive of the idea of drug checking. This view is in line with findings from drug checking literature: a recent assessment of expectations towards prevention at Berlin’s party scene showed that the most demanded preventive measures were drug education and drug checking [39]. However, the destruction of drug samples following analysis was highlighted as a possible risk and disincentive that may impact engagement with the service.

Main learnings: The concept of delivering a Home Office-licensed pharmacist-led drug checking service in an established SMS has been demonstrated here. The service received significant publicity and was positively received.

### 5.1. Obtaining a Home Office Licence

The legislative considerations are crucial when establishing a drug checking service [9,11]. These considerations may vary between countries and depend on whether a drug is controlled under international conventions or whether it is controlled under national legislation or more localised restrictions. In recent years, with the lack of availability of traditional drugs of abuse and the emergence of NPS, drugs were sold in complex mixtures to hinder their detection [36]. Therefore, it became evident that drug checking needs to operate under a licence that enables the handling of Schedule 1 drugs, which may be unclaimed on the product label but present in the “unknown” drug sample. In some services such as Streetwork, the youth advisory service of the city of Zurich, drug possession by the analysts undertaking the drug checking is illegal [4]. In such circumstances, individuals were asked to undertake the drug checking themselves [17], which may impact outcomes. In this pilot, drug samples provided were not returned to their owners following the drug check, in line with UK legislation [28].

### 5.2. Statement of Intent

For the purpose of this pilot, a statement of intent was formulated and written on all pilot-related documentation including the participant information sheet and the consent form. The statement outlined that the organisations involved in this pilot, do not condone drug use, provide confirmatory analysis or state that substances are “safe” to use. This was important, because drug checking has been perceived as a controversial activity as it may be viewed as “legitimising drug use”; however, Ritter (2015) argued that “laws in liberal democracies exist to protect people from harm, not to legislate on matters of personal moral virtue” [40]. In addition, it is also perceived that drug checking may be “promoting drug use”; however, evidence submitted by Brennan and Davidson (2006) to the Parliamentary Joint Committee on the Australian Crime Commission for the purpose of the inquiry into Amphetamines and Other Synthetic Drugs (AOSD) outlined that drug checking and monitoring of the drug market do not promote or increase drug use [41]. These findings were confirmed via a study which explored the public’s perception of drug checking among Twitter users [10]. In addition, an evaluation of The Drugs Monitoring and Information System (DIMS) showed that drug use has remained “stagnant” since the introduction of the service in 1992 in the Netherlands [42].

### 5.3. Maximising the Use of Pharmacists through a Pharmacist-Led Service

The service made use of highly specialised pharmacists in the fields of substance use and NPS. This enabled the provision of a holistic, analytical and clinical approach through appropriate evaluation of the drug checking analysis, review of risk assessments and tailoring of the harm reduction advice. Having a pharmacist on site to conduct the drug checking as well as providing harm reduction interventions was key to this study as pharmacists are equipped with the skills and expertise to identify “red flags” that require immediate referral, or where harm can be reduced from the use of illicit substances, e.g., through providing advice on adverse drug reactions and interactions with regular medicines.

### 5.4. Multi-Disciplinary Assisted Approach in Decision-Making

A multi-disciplinary team enabled the provision of a holistic approach to maximise clinical outcomes. In this respect, the drug checking team was able to use the senior staff at the existing service to aid decision making, for example, when there were potential safeguarding concerns. There were occasions when drug checking was declined, or decisions were made not to disclose the results for ethical reasons. People who were known to the SMS were often keen to involve their allocated recovery worker, who equally reported an added benefit of knowing the drug checking result to complement the harm reduction work that they were conducting with the individuals.

### 5.5. Being Based in an Existing Service Had Benefits

By undertaking the drug checking in an existing SMS, people were offered a much wider level of support and appropriate referrals. The SMS staff could link people to local support groups and to inform users about the different activities and services that the SMS offered. Participants were able to get support from other organisations such as probation, GPs, learning disability, mental health and psychological therapy services, housing, employment and social care because the staff know the area and what is available locally.

### 5.6. Engagement with the Service

Participants attended the service and proactively sought advice and support. Due to perceived stigma, just walking through the door of a SMS may represent a barrier for some users. Individuals who used this service may be viewed as a different cohort to those who may engage with similar services in a music festival or nightclub setting: they are more likely to be taking substances chronically, engage with higher risk activities (such as injecting) and present at various stages of recovery from substances. Even though the pilot ran just over four days, with limited daytime opening hours, advertisement, marketing and recruitment, people still came in and sought access to the service. Individuals who had never accessed the SMS before, only accessed the service after hearing about the service through media coverage. Some users requested that their recovery worker was present during the harm reduction intervention, which meant that it had the potential to be integrated with their treatment plan. This outcome, in turn, was a positive indicator of a functional treatment system with trustworthy and inclusive recovery workers.

### 5.7. Diversity of the Service Users

The service was not a festival setting or hosted within the nighttime economy: it was hosted in an existing community SMS and that affected which users attended and what assistance could be offered. People who came to access the service were from a broad range of ages, backgrounds and in possession of variety of substances, some were already being supported by the SMS, others only used recreationally, as well as family members who were looking for advice and support.

### 5.8. The Technology Worked in a Busy and Public Place

The technique (handheld Raman spectroscopy) has been employed in previous drug checking services [8,11,20]. However, the instrument that was employed in this pilot was enhanced by research-based capability to improve drug detection [29,30,31,32]. This was the first time the research-based technology was trialled in a SMS using bespoke protocols among “real people with real samples” in a busy clinical setting. Throughout the pilot, an iterative approach was taken to refine the processes used. This approach will inform the running of similar services in future. It was proposed that running the pilot did not overly interfere with the normal running of the SMSs and having access to a dedicated area for conducting the checks was an important part of this provision (with appropriate risk assessments in place). The research-based technology enabled a better understanding in “real time” about what people are using and allowed for better tailored harm reduction interventions to be delivered. The in-field analysis showed that the instrument performance was limited in highly coloured and highly adulterated samples, and where the active ingredients were possibly present in small concentrations.

### 5.9. Shared Intelligence

During the pilot, there were no samples which gave cause for concern that necessitated initiation of the alerts cascade; however, on one occasion, intelligence was shared with the local police with consent from the individual.

### 5.10. Awareness of Drug Trends on the Street

A wide range of samples of various solid formulations, herbs and powders were submitted, including suspected heroin, cocaine, amphetamines, LSD, benzodiazepines and synthetic cannabinoid receptor agonists (SCRAs). The smallest amount received and successfully identified was about 2 mg. Analysis of complex mixtures (such as herbs or formulated tablets with potentially high cutting agent content and low psychoactive substance content) was challenging. Suspected adulterants and cutting agents such as taurine, caffeine or lactose were identified. Analysis was successful for highly pure psychoactive substances although purity was not reported to individuals. Analysis time per sample ranged 1–15 min. The actual wait times for service users (how long they had to wait for results of the analysis process) was up to about 30 min. The length of wait times depended on sample manipulation to enhance identification of content and the number of samples. Before disclosing the results, the team discussion included consideration of the likelihood of false positive results and the associated high risks. The synergistic or additive effects of mixture components were also considered, along with the person’s medication history and co-morbidities. It is important to note that drug checking results were mostly identifying contents which were not expected by the user. This finding was true for highly adulterated samples, where users reported that drug appeared “weak”, unlike previous batches that they previously obtained from the same dealers.

### 5.11. Harm Reduction Interventions

In this pilot, various interventions were offered including needle syringe provision, safe storage boxes, naloxone and vaccinations for blood-borne viruses. However, they were not delivered as a direct result of this intervention as, in most cases, they had already been provided by the SMS.

### 5.12. Implications on Drug Policy and Education

The findings from this pilot have informed drug policy nationally and internationally. Nationally, the UK House of Commons. Health and Social Care Committee (2019) drug policy report incorporated a witness statement about our study and highlighted the importance of such harm reduction interventions in acting as an “early warning system” with significant implications on mitigating the wider harms [43]. Internationally, in New South Wales (Australia) [44], in a joint inquest into drug-related deaths at music festivals, Coroner Harriet Graham referred to our study 46 times in the court report and has recommended the continued trialling of drug checking despite it being opposed by the Australian Parliament [45]. Our study was designed within the framework of a Home Office licence and within an established substance misuse service. Findings show that the combination of drug checking with legal, clinical, social and multidisciplinary frameworks has great implications on harm reduction and engagement with services. Despite our limited sample size, positive outcomes were demonstrated. Independent and external feedback from key stakeholders in government, local authorities and service providers have been positive and have led to additional funding in this area. Specialist pharmacist interventions played a significant role in the scientific analysis of the drug products allowing for optimised clinical outcomes for patients particularly those with complicated medical histories. This study bridged the knowledge gap that pharmacist may have on the pharmacodynamics/pharmacokinetics of psychoactive substances and along with specialist substance misuse knowledge and competencies and multidisciplinary team working have set the foundation for holistic and tailored patient-centred care. In our previous research, we made recommendations on designing specialist curricula to enhance knowledge of healthcare professionals of psychoactive substances [46].

### 5.13. Limitations and Future Work

The timeframe allocated for this pilot was too short owing to logistics, availability of resources and costs. The pilot ran during the daytime and on weekdays only, which may have limited the number of individuals who wished to access the service (for example if in they were in full employment). Recruitment was challenged by other barriers including local elections. The screening technique used lacked accuracy (false positives negatives) specially with highly coloured and highly adulterated samples. Feasibility of the study was limited by the geographical location, potential accessibility barriers for Black, Asian and minority ethnic (BAME) and exclusion of people under 18. Evaluation of the analytical methods used for drug checking is subject to future research. The service was anonymously conducted which has restricted follow ups to measure impact of the interventions. To validate and more robustly determine and evaluate the impact of a drug checking service in this context, more data are required. It is proposed that running a much larger study in an urban SMS is needed. There has been significant interest from commissioners, academics, peers, volunteers and other interested organisations. Further research is still required to improve the technologies employed to enhance drug detection.

## 6. Conclusions

Despite the limited number of days of this pilot and the low drug sample numbers, the findings demonstrated the proof-of-concept that a pharmacist-led Home Office-licensed drug checking service can be successfully implemented in a community SMS. Drug checking is not a simple process and can be implemented using several different analytical methods that vary widely in cost, portability, speed, sensitivity, specificity and accuracy. Each method has its own inherent limitations and suitability based on the setting and prevalence of certain types and forms of drugs. In a SMS, this drug checking pilot has demonstrated improved accessibility to a wide variety of people, including people with high risk use behaviour such as injection drug use, men who have sex with men (MSM), and people who enter substance use disorder treatment services, opiate and crack cocaine users (OCUs), individuals supplementing their drug use with NPS or prescriptions drugs, and not just people who go clubbing/attend festivals. Our pilot, operating from a community SMS (Addaction’s site in Weston-Super-Mare) enabled a service to be offered to chronic users, highly marginalised and stigmatised individuals, including people who experience homelessness and people accessing needle syringe provision. Complexity of the drug sample and variety of substances used per individual had a great impact on drug checking analysis and tailoring of the advice and intervention. A pharmacist-led service has demonstrated the feasibility of delivering a holistic pharmaceutical care approach coupled with harm reduction advice relating to drug use.

As pharmacists, we consider drug checking to be consistent with the principles of harm reduction and support further trials to inform the role of drug checking within the UK. We believe they should occur in controlled environments where relevant evidence-based support and referral signposting can be performed. This approach is in line with the UK drug strategy and commitment to harm reduction with the aims to reduce demand, restrict supply, and build recovery capital. There is a huge need for services to engage and work with a new, younger generation of people who use substances. We believe pharmacists can play a significant role in reducing harms from illicit substance use through drug checking. In line with the General Pharmaceutical Council’s standards for pharmacy professionals, pharmacists have a duty of care and relevant competency to provide patient-centred care and support ensuring harm prevention and reduction and access to health care. 

The benefits of drug checking are not about the “check” itself, but proactively engaging people who use substances, creating opportunities for experienced healthcare professionals to have a dialogue with users, engage people in treatment, design and formulate personalised support to prevent and reduce associated harms. Drug checking can contribute to early warning systems on emerging threats related to substance use. It should not condone drug use or encourage substance use: it should be non-judgmental and professional and invite people to become partners in a dialogue to prevent and reduce harms if they insist on using the drug even after knowing the likely content in the sample. In the context of pharmacovigilance, drug checking can provide timely data to various stakeholders including people using substances, police, national health systems and policy-makers. This model has been shown to be appreciated by service users who feel they can trust the information provided in a safe environment, which provides education, advice and support. In many situations, users do not know the real content of the drug and may be anxious, especially if they have purchased the drug from a new source, which may also result in increased willingness to sacrifice part of their drugs for checking and subsequent behavioural change and engagement with the service.

Recent drug trends have shown a number of risk factors including: a wide range of inter- and intra-batch variation, modern adulterants, higher production skills, idiosyncratic effects, poly-substance use and high accessibility to “bad batches”, which are widely distributed and consumed prior to testing. Marginalised populations with higher rates of addiction cannot usually wait to get substances tested. Drug checking services are one of many tools that can be employed to prevent and reduce harms, raise alerts, monitor drug displacement and drug trends in real-time. Furthermore, education and training are crucial to foster open dialogues about drug use and drug harms, to enable the development of skills and changes in behaviour, which can prevent harms related to the use of substances. Further research is needed to link user reports of desired undesired effects as well as signs and symptoms of toxicity coupled with analysis findings. Additionally, there may be a significant role for the Internet in reaching out for people and identifying targeting risk behaviours and improving non-judgmental messaging to remove stigma that prevents vulnerable people from accessing treatment.

## Figures and Tables

**Table 1:** Summary of questionnaire findings.

ID	#011	#012	#013	#014	#015	#016	#017-8	#020	#021-3	#024
**Age**	34	55	28	46	50	51	17	38	40	28
**Gender**	Male	Male	Male	Male	Female	Male	Male	Female	Female	Male
**Employed**	No	No	No	No	No	Yes	No	Yes	No	Yes
**Physical Health concerns**	No	No	No	No	Yes	No	Yes	No	No	No
**Mental health concerns**	No	Yes (prescribed medication)	Not disclosed	No	Yes	No	Yes (medication under review)	Yes (associated with recreational drug use)	Yes (prescribed medication)	Yes (associated with recreational drug use)
**Current use of alcohol**	No	No	Yes (not dependent)	Yes (dependent)	No	Yes (not dependent)	Yes (not dependent)	Yes (not dependent)	Yes (dependent)	Yes (not dependent)
**Other illicit substances currently using**	Cocaine Spice	None disclosed	Cocaine	Heroin Cannabis Crack Diazepam	Heroin Cannabis Amphetamine Ecstasy Temazepam	None	Cannabis	Cocaine	Cannabis Amphetamine Methamphetamine Diazepam Alprazolam	Cocaine Cannabis Ecstasy
**Medication currently prescribed/bought over the counter?**	Methadone (supervised consumption)	Quetiapine Propranolol Mirtazapine Buprenorphine (supervised consumption)	None	Methadone (supervised consumption)	Anticoagulant Statin	Citalopram	None	None	Pregabalin Mirtazapine	None
**Previously overdosed?**	Once—accidentally on painkillers	Twice—many years ago	Not disclosed	Twice	Once – last week	No	No	No	Twice—many years ago on prescribed medication	No
**Contact for support with substance use?**	Current psychosocial and prescribed via Addaction, probation, Narcotics Anonymous	Psychosocial and prescribed via Addaction	Not disclosed	Psychosocial and prescribed via Addaction	Re-engaged today- psychosocial via Addaction	No	No	No	Psychosocial via Addaction	Not currently (previously attended Cocaine support)

**Table 2:** Summary of results about the relinquished sample and harm reduction intervention(s).

ID	#011	#012	#013	#014	#015	#016	#017-8	#020	#021-3	#024
**Details of sample**	SPICE smoked. From street dealer. Own use. Effects as expected *“super strong”*	VALIUM swallowed. From street dealer. Own use. Felt drowsy *“like weak diazepam but varies”*	COCAINE Suspected dealing	HEROIN smoked. From street dealer. Own use. Little effect	“CALMER” swallowed. Own use. Effects as expected, felt calmer and less anxious	COCAINE snorted. From friend/family Used by partner. Unknown effects	LSD dissolved on tongue. VALIUM swallowed. From street dealer/friend. Own use. Unknown effects (have not tried yet).	COCAINE snorted. From street dealer. Sharing with friends. Bad headaches (which has prompted the check)	AMPHETAMINE swallowed (little effect). DIAZEPAM swallowed. XANAX swallowed, bought online. Own use.	ECSTASY swallowed. From a friend/family. Sharing with friends. Unknown effects (have not tried yet)
**Summary of harm reduction advice and signposting provided**	Overdose awareness (including polypharmacy), dosing and how to reduce down nb complications previously so awaiting inpatient detoxification. Continued working with Addaction, Narcotics Anonymous and probation.	Overdose awareness (including polypharmacy), dosing, variability in batches, and how to reduce. Feels their recovery worker has already talked about harm reduction lots. Continued working with Addaction.	Signposted to local learning disability services and carer support group. Harm reduction advice given including use of paraphernalia in the home and cocaethylene.	Requested friend and recovery worker to join the consultation. Overdose awareness (including polypharmacy) and smoking cessation. Safe storage and naloxone recently discussed with recovery worker. Continued working with Addaction.	Requested recovery worker to join the consultation. Overdose awareness (including polypharmacy), management of underlying anxiety, dosing and how to reduce down nb history of seizures. Safe storage, naloxone (including supply for daughter) and BBV risks recently discussed with recovery worker/prescriber. Signposted to local mental health services/local psychological therapies. Continued working with Addaction.	Reported no concerns about current recreational use. Alcohol brief intervention including choice of drink, interaction with alcohol/cocaethylene, practical steps for reducing use including. less often, shorter lines, QT prolongation, Sharing advice with partner and signposting to support.	Accompanied by adult friend. Plans to use substances to manage mental health symptoms after lack of success with conventional treatment. Discussed safe environment, lack of evidence base, mental health management including signposted to GP (referring to CAMHS), local young persons and drug service (from another area). Alcohol brief intervention including reducing alcohol intake when binging. Offered time with Addaction Consultant Psychiatrist if able to return (did not return).	Reported no concerns about current recreational use. Alcohol brief intervention including choice of drink alternating with non-alcoholic drinks, interaction with alcohol/cocaethylene, practical steps for reducing use including less often, shorter lines, breaks between binges. Discussed likely cause of headaches, variability in batches/risks of unknown content, BBV risk and management including sharing equipment.	Only returned for amphetamine result. Already aware of risks associated with stimulants. Overdose awareness (including polypharmacy), management of underlying anxiety, sleep hygiene including new medication. Significant history of substance misuse and notable progress with recovery. Signposted to local mental health services/local psychological therapies. Continued working with Addaction.	Reported no concerns about current recreational use but aware that drug use affects mental health and has strategies for this. Discussed self-regulation of cocaine/alcohol and interaction/cocaethylene, variability in batches/risks of unknown content. Already aware of risks including not using alone, using when riding bike/working with machinery.
**Doing anything differently?**	No—awaiting rehab and already knows the service well	No—already aware of risks and already knows the service well	Significant improvement in understanding of the service	Somewhat improved understanding of the service. Going to take less and plans to buy less often	Plans to reduce from twice a day to once a day but prioritising reducing use of heroin as this is only occasional. Already knows the service well	Hope to reduce and stop. Somewhat improved understanding of the service	Plans to no longer take. Somewhat improved understanding of the service (Addaction is not their local or young person’s service)	Plans to take less and less often and not share paraphernalia. Will also tell friends. Now aware of the service should drug use be an issue in the future	Plans to take less often. Prioritising reducing use of crystal meth first. Already knows the service well	Plans to take things slower and more carefully especially with alcohol and reduce alcohol intake when using
**Comments on the service**	Heard about via Probation. No concerns	Heard about via friend/family. Questioned anonymity as known by staff	*“Very grateful”*	Heard about via Addaction staff. No concerns	Heard about via Doctor. *“It’s a great idea”*	Heard about via TV. *“It’s a very good idea”*	Heard about via friend/family. *“A good idea as concerned about honesty of dealer”*	Heard about via TV. *“Good thing is remains confidential”*	Heard about via Addaction staff. No concerns	Heard about via the internet. *“It is a good service for the community. There should be more testing facilities around”*

**Figure A1:**
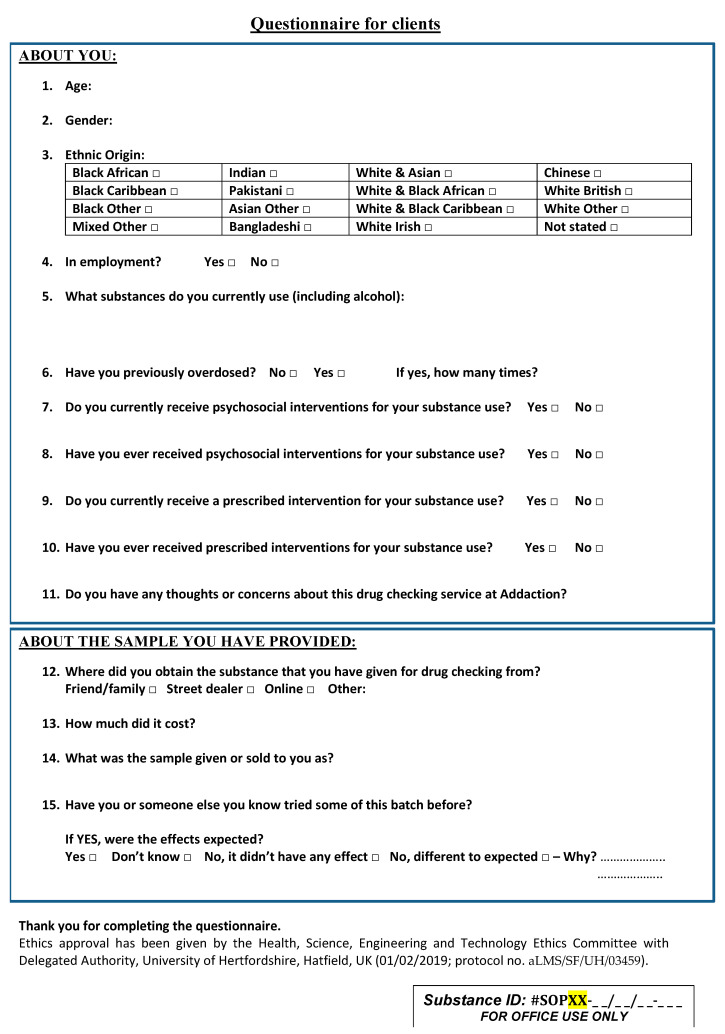
Questionnaire for clients, completed by the client, whilst awaiting the result of their sample analysis and used when delivering the tailored harm reduction intervention.
